# Blue lighting accelerates post-stress relaxation: Results of a preliminary study

**DOI:** 10.1371/journal.pone.0186399

**Published:** 2017-10-19

**Authors:** Jesus Minguillon, Miguel Angel Lopez-Gordo, Diego A. Renedo-Criado, Maria Jose Sanchez-Carrion, Francisco Pelayo

**Affiliations:** 1 Department of Computer Architecture and Technology, University of Granada, Granada, Spain; 2 Research Centre for Information and Communications Technologies (CITIC), University of Granada, Granada, Spain; 3 Department of Signal Theory, Telematics and Communications, University of Granada, Granada, Spain; 4 Nicolo Association, Churriana de la Vega, Spain; 5 School for Special Education San Rafael, San Juan de Dios, Granada, Spain; University of Pennsylvania, UNITED STATES

## Abstract

Several authors have studied the influence of light on both human physiology and emotions. Blue light has been proved to reduce sleepiness by suppression of melatonin secretion and it is also present in many emotion-related studies. Most of these have a common lack of objective methodology since results and conclusions are based on subjective perception of emotions. The aim of this work was the objective assessment of the effect of blue lighting in post-stress relaxation, in comparison with white lighting, by means of bio-signals and standardized procedures. We conducted a study in which twelve healthy volunteers were stressed and then performed a relaxation session within a chromotherapy room with blue (test group) or white (control group) lighting. We conclude that the blue lighting accelerates the relaxation process after stress in comparison with conventional white lighting. The relaxation time decreased by approximately three-fold (1.1 vs. 3.5 minutes). We also observed a convergence time (3.5–5 minutes) after which the advantage of blue lighting disappeared. This supports the relationship between color of light and stress, and the observations reported in previous works. These findings could be useful in clinical and educational environments, as well as in daily-life context and emerging technologies such as neuromarketing. However, our study must be extended to draw reliable conclusions and solid scientific evidence.

## Introduction

Light has an essential role in our ecosystem. For example, plants use the energy of sunlight to live through the photosynthesis process. Light is also vital for many other living beings including humans. Chromotherapy, also named cromatherapy, colorology or therapy of colors, is an old alternative medicine method that uses the energy of electromagnetic radiations in the visible spectrum (i.e., colored light) to produce changes in the human body [1]. Although therapy of colors is not well-described and frequently considered pseudoscience, a number of studies have tried to explain the effects of colors on the human body. Some of them have focused on physiological and others on emotional changes. Next two paragraphs elaborate on these two aspects.

On the one hand, several studies have investigated the influence of color of light on human physiology throught biochemical markers such as cortisol [2,3] or melatonin [2–4] level, and bio-signals such as electrocardiographic (ECG) [3,4] or electroencephalographic (EEG) [3,5–11] signals. In this sense, only a few colors have been investigated and blue is in most of the studies. It has been proved that turquoise (a variant of blue) light is an effective way to treat jaundice in newborns [12]. Long exposures (several hours) to blue light provoke melatonin suppression and phase shifting in the circadian system with sleepiness reduction and alertness augmentation [4,5,13,14]. Light can modulate alertness-related subcortical activity, thus stimulating cortical activity not involved in visual cognitive processes [15]. A recent study suggested that early EEG responses (e.g., event-related potentials during the first milliseconds) depend on their adaptation to different colors of light [11]. Another recent and preliminary work showed that a short stay (20 minutes) inside a blue room caused cortisol level reduction in a woman [3].

On the other hand, some colors have been related to emotions. For example, different hues were linked to different pleasure and arousal levels [16]. Lighting was demonstrated to affect the mood of elderly people [17]. A study about the influence of color of walls in learning environments proved that pale colors caused more relaxation than vivid colors, and that heart rate decreased with short-wavelength colors (e.g., violet, blue and green) in comparison with longer-wavelength (e.g., yellow and red) [18]. In addition, a few authors have successfully treated people with behavior disorders by influencing their emotional states (e.g., causing mental calm) by color lighting. For instance, pink light was successfully utilized to reduce aggressiveness of delinquents in prison [19]. Furthermore, another color-lighting-based method with blue light have been used for disruptive behavior disorders in the School for Special Education San Rafael, Granada (Spain) with substantial improvements [20]. However, these emotion-related studies, with a few exceptions [21], have a common lack. Methodology, results and conclusions were based on empirical and, in some cases, subjective observations [22,23]. This lack of methodology reduces the ability to reproduce the results. Objective information obtained by a methodological procedure is much more powerful than reported subjective feelings [24]. The way to assess emotions through a methodological procedure is still an open question to address.

In the literature, there are recent examples of rigorous procedures to recognize emotions based on bio-signals such as EEG or ECG [25] [26]. Specifically, several authors have demonstrated that stress is reflected by changes in brain rhythms measured at frontal cortical areas [27–29]. The Relative Gamma (RG) power, which is a power ratio between brain rhythms (see section EEG signals for further details), is suitable for that purpose. In fact, it has been previously utilized in meditation-relaxation [30,31] and stress [32] studies. In addition, the heart rate (HR) is, under certain conditions, commonly accepted as stress marker [33–37]. Also, brain imaging techniques such as functional magnetic resonance imaging (fMRI) [38,39], near-infrared spectroscopy (NIRS) [40] and positron emission tomography (PET) [41] have been applied with the same or similar purpose.

For a better control of the conditions under which stress is measured, various techniques have been developed. For instance the Montreal Imaging Stress Task (MIST) [42]. The MIST is a well-described method to cause stress in humans with a methodological procedure [36]. It induces mental arithmetic load together with psychosocial stress. It has been used in various stress-related works [38,39,41,43]. Finally the use of a time-out room or a specific chromotherapy room provides the enough level of isolation to perform stress-related experiments with environmental condition under control.

The aim of this pioneering study was the objective assessment of the effect of blue lighting (test group) in post-stress relaxation, in comparison with white lighting (control group), by means of biosignals and standardized procedures. In particular, we used technics and features detailed in the previous paragraph, namely the stress markers RG, HR, a stressing procedure (i.e., the MIST) to elicit a similar initial level of stress in the participants, and the same time-out room used in [20] to guarantee a successful relaxation with stimulus and environmental factors under control. We have designed a reproducible experiment that avoids conclusions based on subjective observations. The results were compared with those of conventional-white lighting and practical implications were inferred.

## Methods

### Experimental design

#### Participants

Twelve healthy volunteers (age range of 18–37 years, mean age of 25.3 ± 4.8 years) participated in the study. Apart from age, no other baseline demographic characteristics were recorded. The participants were recruited during the month prior to the beginning of the study. They voluntarily contacted the research team to participate and were not paid for that. No participant was excluded from the study. The participants declared no experience in EEG or stress-related experiments. They were instructed not to take stimulants or relaxants during 24 hours prior to the experiment. The protocol and informed consent were approved by the Bioethics Committee of the University of Granada (see S1 File). The participants provided their written informed consent to participate in the study.

#### Experimental procedure

Once the informed consent was understood and signed by the participants, they dressed in white hospital uniforms and were equipped for EEG and ECG recordings (datasets available in S2–S13 Files). They were randomly assigned to two experimental groups G1 (test group) and G2 (control group), therefore groups of six participants. Thereupon a stress session was conducted. During that session, all participants performed an adapted version of the MIST. As mentioned, the MIST is a well-described method to cause stress in humans. The goal of this session was to elicit a uniform level of stress in all participants of this experiment. After a training period of 3 minutes, the MIST lasted 6 minutes.

Afterwards, a relaxation session was conducted by using either blue or white lighting within the chromotherapy room. This session was divided into two consecutive blocks of 10 minutes each (i.e., B1 and B2), with the only difference of the color of the light projected in the room. The color sequence was blue-white for G1 and white-blue for G2. During their stay, the participants were monitored by a video camera for safety and artifacts removal purposes.

**Fig 1:**
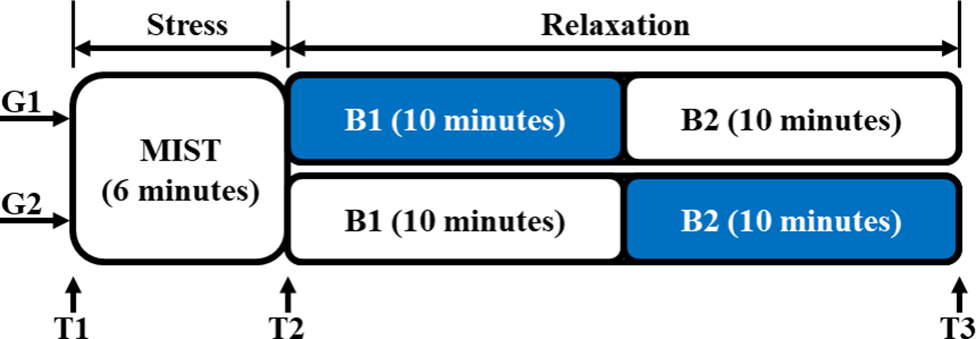
Timeline of the experiment. Both groups G1 and G2 performed the MIST, which lasted 6 minutes. Afterwards, the relaxation session was conducted within the chromotherapy room. This session was divided into two consecutive 10-minute blocks B1 and B2. The color sequence of B1-B2 was blue-white for G1 and white-blue for G2. Three oral tests were taken by the participants before the MIST (T1), after the MIST (T2) and at the end of the experiment (T3).

In order to assess the self-perception of stress, oral tests were taken by the participants three times during the experiment. In particular, the same test was repeated before the MIST (T1), after the MIST (T2) and after the relaxation session (T3). The timeline of the experiment is displayed in Fig 1.

### Experimental setup

One ECG electrode was placed on the non-dominant wrist of the participants. Seven EEG electrodes were placed at Fp1, Fp2, Fz, F3, F4, F7, F8 positions of the 10–20 International System. These positions have been used in reports of successful studies on stress [27–29]. All the electrodes were referenced and grounded to the left ear lobe. The impedance of the electrodes was below 30 KΩ. This value is much lower than the input impedance of the acquisition system and it is enough to guarantee an insignificant degradation of the recorded signals. EEG and ECG signals were recorded at 540 Hz with the Miniature Data Acquisition System of Cognionics (Cognionics, Inc., USA).

The MIST was conducted within a classroom. A graphical user interface (GUI) of the MIST was implemented in Matlab R2014a (The MathWorks, Inc., USA). During the task, the participants were sat on a chair while they played the Matlab-based GUI using the touchpad of a laptop. In order to avoid severe artifacts in EEG and ECG signals, they were instructed to exclusively move their dominant hand using the touchpad.

**Fig 2:**
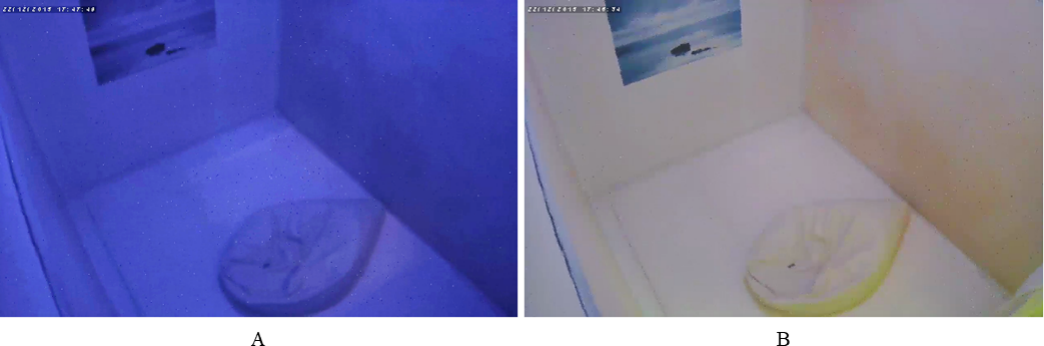
Chromotherapy room. (A) Chromotherapy room with blue lighting. (B) Chromotherapy room with white lighting.

During the relaxation session, the participants stayed laid on a comfortable puff-shaped seat placed inside a 6 m2 chromotherapy room. This room was specially designed for relaxation and has been used in the school as time-out room for children with behavior disorders. The walls were compounded by a white padded material. The illumination system consisted of three sets of light-emitting diodes (LEDs): red (616 nm wavelength and 2.19 cd/m^2^ luminance), green (550 nm wavelength and 4.02 cd/m^2^ luminance) and blue (471 nm wavelength and 1.37 cd/m^2^ luminance) LEDs. White light (similar to typical office room light) was generated by powering up all the LEDs. Blue light was generated by powering up the blue LEDs with red and green LEDs powered down. Wavelength and luminance were measured by the i1 Display Pro calibration device (X-Rite, Inc., USA). The chromotherapy room is displayed in Fig 2. The participants were instructed not to close their eyes (except for blinking) and to avoid moving or gazing any part of the room (i.e., the thousand-yard stare) during the relaxation session.

The oral test for assessment of the subjective self-perception of stress was based on the Spanish version of the Perceived Stress Scale (PSS) [44]. Only one question was analyzed in this paper: *If 0 is the minimum level and 4 is the maximum level*, *what is your stress level*? The third test T3 included the following extra question: *Which color*, *blue or white*, *have you felt more relaxed with*?

### Signal processing

#### EEG signals

Recorded EEG signals were bandpass filtered (1–100 Hz) using a second order Butterworth IIR filter. A notch filter was applied to remove power-line couplings. Ocular artifacts were removed using independent component analysis. It was performed by using the EEGLAB Matlab toolbox (Swartz Center for Computational Neuroscience, USA).

A spectral analysis was applied to the preprocessed data of each subject. Two-second epochs (no overlap) were extracted, z-scored and then the power spectral density (PSD) estimated for each EEG channel. The mean power at different frequency bands was calculated through the PSD and then averaged across all channels. The RG was computed as described in (1). It corresponds to the power ratio between Gamma rhythm (25–45 Hz) and the slow rhythms Theta (4–7 Hz) and Alpha (8–13 Hz). These spectral features were used in previous emotion-related works [25,30–32].

$$RG=Power_{25-45\;Hz}/Power_{4-13\;Hz}$$ (1)

Then the RG were interpolated (inter-participant time warping), smoothed with a moving average filter (40 samples), z-scored and then averaged across the participants of each group, G1 and G2. In the MIST, the results were averaged across all the participants (i.e., G1 plus G2) since the task and the experimental conditions were the same for groups.

#### ECG signals

The recorded ECG signal was bandpass filtered (4–24 Hz). A second order Butterworth IIR filter was used to enhance the R-peak of the QRS complex [45]. The HR was computed every 30 seconds with 90-second epochs (66% overlap) by estimating R-peak intervals with an automatic procedure. The HR was interpolated (inter-participant time warping), smoothed with a moving average filter (2 samples), z-scored and then averaged across the participants of each group, G1 and G2. As it was done for the RG, the HR was averaged across all the participants in the MIST.

### Statistical analysis

The mean and the standard error of the mean (SEM) of the subjective self-perceived stress level were estimated from the answers to tests in T1, T2 and T3. The one-way ANOVA test was applied to compare answers of groups G1 and G2. The Kolmogorov-Smirnov (KS) test was used to assess normality.

The SEM was also computed for the RG and the HR. In order to simplify the analysis, RG and HR plots were divided into adjacent segments corresponding to linear trends (i.e., linearized RG and linearized HR, respectively). The first segment (i.e., Seg1) corresponds to the MIST (from minute 0 up to the point of maximum RG or HR within the transition time interval between the MIST and B1). This segment is shared by all groups since all the participants were averaged together in the MIST. The second segment (i.e., Seg2) ends at the point matching with the first minimum of RG or HR. The third segment (i.e., Seg3) ends at the second minimum of RG. The fourth segment (i.e., Seg4) ends at minute 16 (transition from B1 to B2). In case of the HR, Seg3 and Seg4 were merged into one segment (i.e., Seg3). The last segment (i.e., Seg5 of RG and Seg4 of HR) ends at minute 25, that is, one minute before the end of B2 (the last minute of B2 contained residual data from processing, thus it was omitted). These segments were fitted to a line by simple linear regression. The goodness of the fit was evaluated by means of R^2^. For each segment, the slopes of G1 and G2 were estimated. The slopes are numerical indicator of the rate of decreasing of stress level that we use to compare the effects of blue and white lighting during the relaxation session. The null hypothesis that both slopes were the same was checked by estimating the Student’s t statistic on N-4 (N is sample size) degrees of freedom. Student’s t statistic was computed using (2), where *b*_*1*_ is the slope 1, *b*_*2*_ is the slope 2 and *SE*_*b1-b2*_ is the standard error of the difference.

$$t=(b_{1}-b_{2})/SE_{b_{1}-b_{2}}$$ (2)

In order to estimate the time instants of zero RG from the linear regressions, zero-crossings of some segments were computed. For that, the equation of the regression (i.e., *y* = *bx* + *a* where *b* is the slope and *a* is the intercept) was evaluated at y = 0.

Finally, the RG was averaged minute-by-minute (from the beginning of the relaxation session). Then an inter-group comparison was performed by the Kruskal-Wallis (KW) test. Relative gamma is the ratio of the power of frequency bands of EEG signals. None of these terms follow a normal distribution and nor the ratio. Thus, the RG was not expected to follow a normal distribution. In fact, data did not pass the normality test Kolmogorov-Smirnov (KS) (p-value > 0.05). Similar results were obtained for the HR (p-value > 0.05). In this situation ANOVA could not be applied since it requires normally distributed data. For this reason the Kruskal-Wallis test was chosen. This non-parametric test is utilized to check if two datasets come from the same distribution. It can be used as an alternative to the ANOVA test when the distribution cannot be assumed to be normal. For all the statistical tests of this paper, the significance level was set at α = 0.05.

## Results

### Subjective self-perception of stress

**Fig 3:**
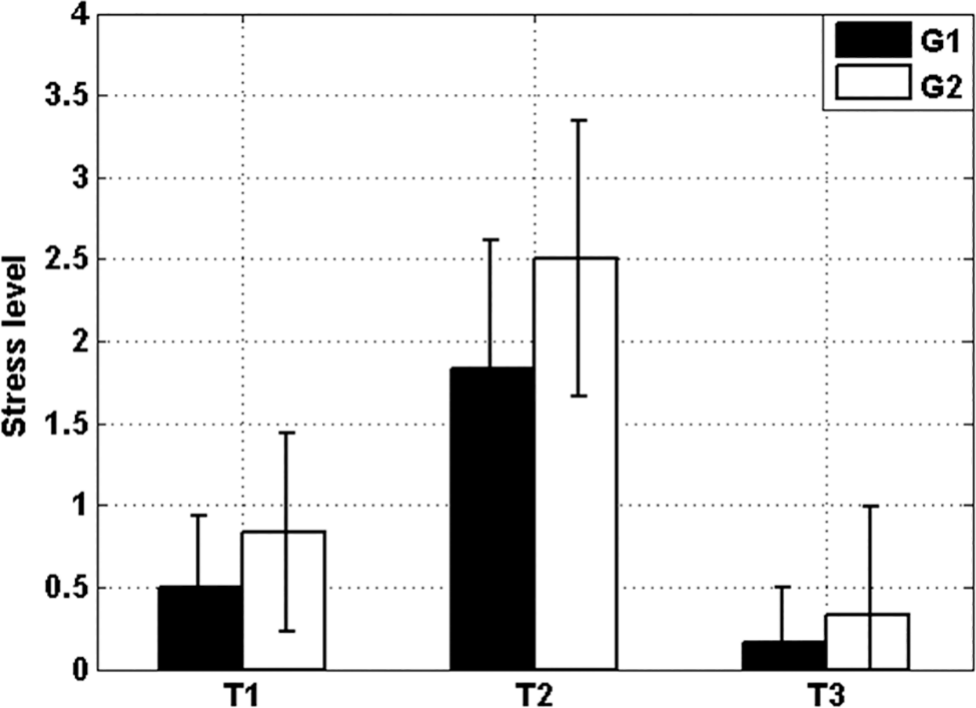
Mean (bars) and SEM (errorbars) of the subjective self-perceived stress level. G1 (black) and G2 (white). At each time (T1, T2 and T3) there was no significant inter-group difference. The intra-group analysis reveals significant differences of subjective stress level T1-T2 and T2-T3 for both groups. The latter proves that both the stress and relaxation sessions were satisfactory completed.

The mean and SEM of the answers to the question asked to G1 and G2 in T1, T2 and T3 are displayed in Fig 3. In T1, T2 and T3, G2 reported more self-perceived stress. The ANOVA test did not disclose statistically significant inter-group differences in T1 (p-value = 0.40), T2 (p-value = 0.28) and T3 (p-value = 0.66). However, the same test found significant intra-groups differences. For G1: T1-T2 (p-value = 0.02) and T2-T3 (p-value = 0.00); for G2: T1-T2 (p-value = 0.01) and T2-T3 (p-value = 0.00). Regarding the extra question asked in T3 about the light which causes more relaxation, 10 out of 12 participants (83% with confidence interval [55, 95] %) answered that they felt more relaxed with the blue light.

### Frontal relative gamma and heart rate

This section shows the results obtained from the processing of EEG and ECG signals.

**Fig 4:**
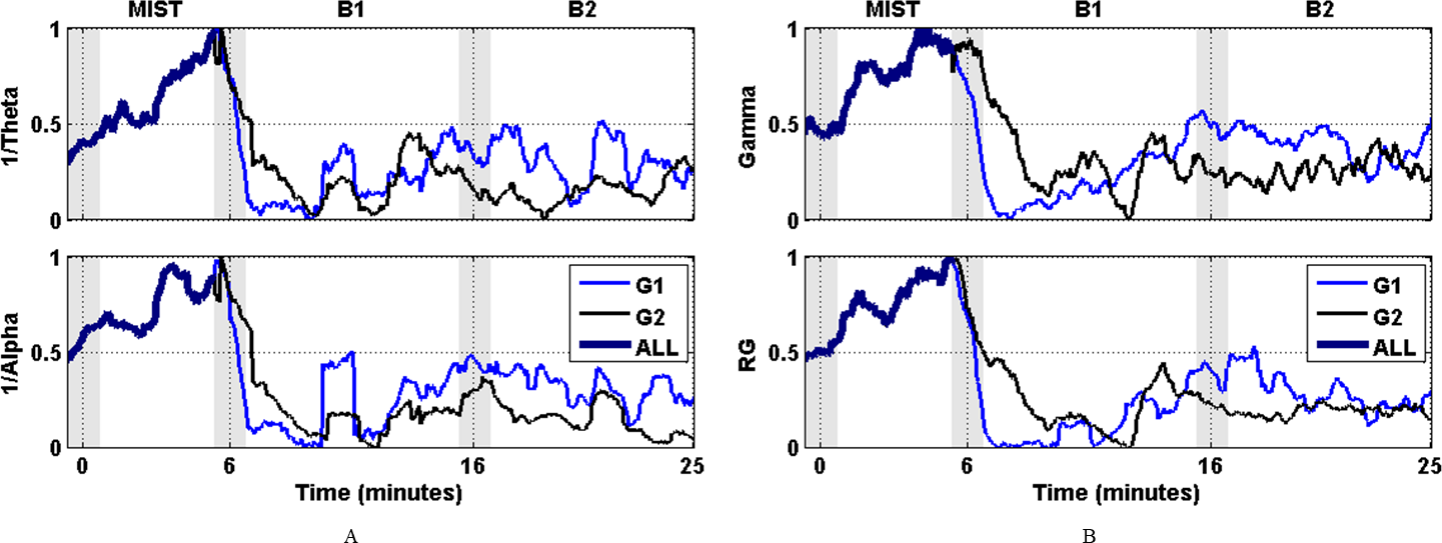
Normalized mean spectral power of G1 (blue) and G2 (black). Shaded bars indicate transition time intervals due to smoothing and interpolation. (A) Gamma power on the top and the RG at the bottom. (B) 1/Theta power on the top and 1/Alpha power at the bottom. The four plots show that despite they exhibit a high level of correlation and similar envelope, the RG computes a smoother version that emphasizes the differences of curves of G1 and G2 during the transition to B1.

The mean inverse power at frequency bands Theta and Alpha, and Gamma and RG power for both groups G1 and G2 are displayed in Fig 4. Shaded bars at T1, T2 and T3 indicate the fuzzy boundary between the stress session (MIST) and blocks of the relaxation session (B1 and B2) due to smoothing and interpolation of epochs during signal processing.

**Fig 5:**
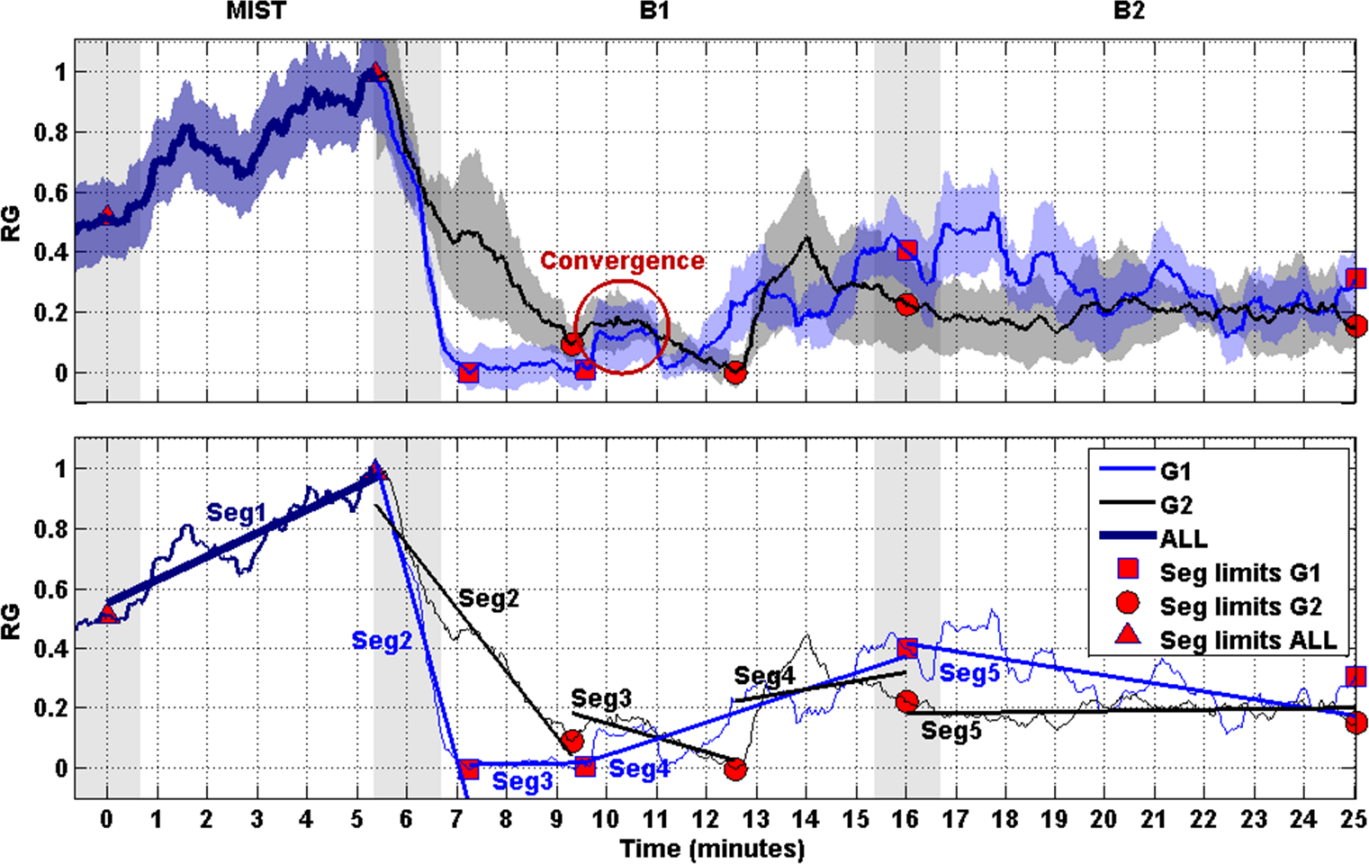
RG and segments. Upper: Curves represent the normalized RG of G1 (blue) and G2 (black). The SEM of the RG is displayed behind the RG curves. Shaded bars indicate transition time intervals due to smoothing and interpolation. The red circumference indicates the time period in which the curves of both groups converge. Bottom: The curves of the upper plot are simplified by their respective linear trends (linearized), thus given rise to segments (i.e., Seg1, Seg2, Seg3, Seg4 and Seg5). Red markers indicate limits of the segments.

The mean and the SEM of the RG of both groups G1 and G2 are shown in Fig 5 (upper plot). For sake of clarity, five segments were regressed on the RG curves and presented in Fig 5 (bottom plot, see section Statistical analysis for a detailed explanation). Table 1 shows the initial and end time and slopes of the segments. The table also shows the goodness of the fit (R2) and the comparison of slopes. Asterisks indicate statistically significant difference (p-value<0.05).

**Table 1:** Information about the linear regression of segments of the RG.

Segment	Initial time (min.)	End time (min.)	Slope (min^-1^)	R^2^	t statistic	p-value
Seg1 ALL	0.0	5.4	0.08	0.82		
Seg2 G1	5.4	7.2	-0.61	0.97	25.96	0.00*
Seg2 G2	5.4	9.3	-0.21	0.93		
Seg3 G1	7.2	9.6	0.00	0.02	13.97	0.00*
Seg3 G2	9.3	12.6	-0.05	0.70		
Seg4 G1	9.6	16.0	0.05	0.72	2.76	0.01*
Seg4 G2	12.6	16.0	0.03	0.07		
Seg5 G1	16.0	25.0	-0.03	0.53	17.59	0.00*
Seg5 G2	16.0	25.0	0.00	0.04		

* indicates statistically significant difference (p-value<0.05).

Table 2 shows the time instants of zero RG. They were estimated from the linear regressions of Fig 5 bottom (see section Statistical analysis for a detailed explanation). The first zero for G1 and G2 corresponds to the zero-crossing of the fitted line of Seg2. The second zero for G1 corresponds to the zero-crossing of the fitted line of Seg4. The second zero of G2 corresponds to the zero-crossing of the fitted line of Seg3.

**Table 2:** Time instants of zero RG.

Zero-crossing	Time (min.)	Time from B1 (min.)
Seg2 G1	7.1	1.1
Seg2 G2	9.5	3.5
Seg4 G1	9.2	3.2
Seg3 G2	13.2	7.2

**Fig 6:**
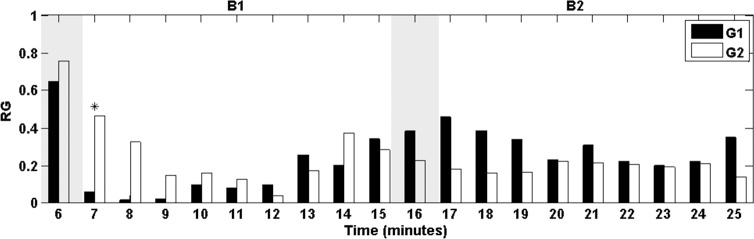
Normalized RG averaged minute-by-minute (from the beginning of the relaxation session). G1 (black) and G2 (white). Shaded bars indicate transition time intervals due to smoothing and interpolation. Asterisks indicate statistically significant difference (KW; p-value<0.05).

Fig 6 shows the values of the RG of both groups G1 and G2 minute-by-minute during the relaxation session. Differences were analyzed by means of the KW test. Asterisks indicate statistically significant difference (p-value<0.05).

**Fig 7:**
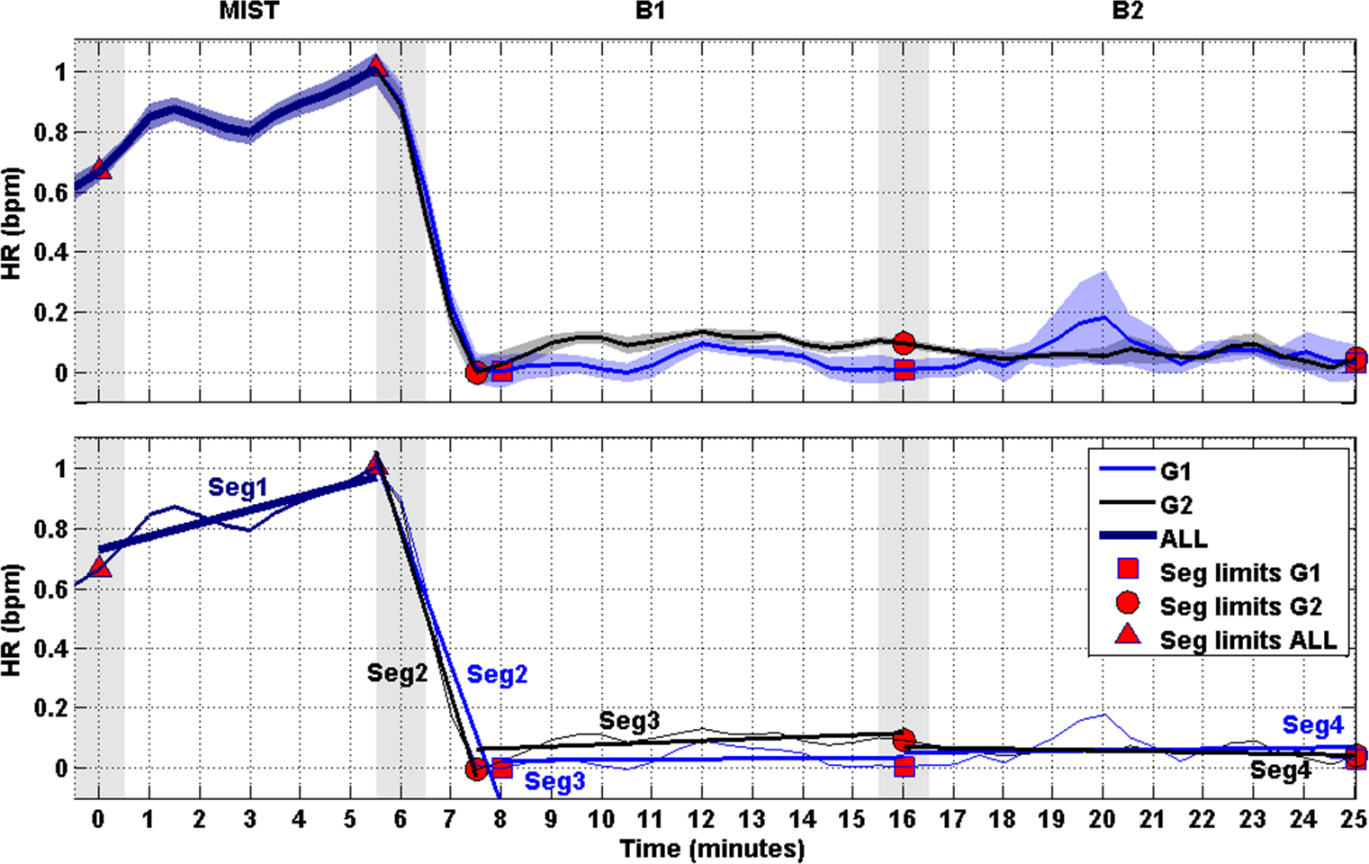
HR and segments. Upper: Curves represent the normalized HR of G1 (blue) and G2 (black). The SEM of the HR is displayed behind the HR curves. Shaded bars indicate transition time intervals due to smoothing and interpolation. The red circumference indicates the time period in which the curves of both groups converge. Bottom: The curves of the upper plot are simplified by their respective linear trends (linearized), thus given rise to four segments (i.e., Seg1, Seg2, Seg3 and Seg4). Red markers indicate limits of the segments.

The mean and the SEM of the HR of both groups G1 and G2 are shown in Fig 7 (upper plot). As it was done before for the RG in Fig 5, segments were regressed on the HR curves and presented in Fig 7 (bottom plot).

## Discussion and conclusions

The results reported in the previous section suggest that color of light influence the relaxation process after the stress session. Specifically, the presence of blue lighting accelerates the reduction of stress level in comparison with conventional white lighting. In our experiment a reduction of more than three minutes (1.1 vs. 3.5 minutes) was achieved with the blue lighting till level of stress converged in both groups. Furthermore, the minimum level of stress remained stable longer with the blue than with the white (3 minutes vs. less than one minute respectively). Although it could seem a small fraction of time, these findings could mean a significant change in the way that time-out rooms are used in episodes of behavior disorders. See section Practical implications and future works for a short discussion of their practical implications.

### Subjective self-perception of stress

Fig 3 shows no significant differences in the level of self-perceived stress between groups at the beginning (T1), after the stress session (T2) and at the end of the experiment (T3). This is an expected result since participants were randomly assigned to the groups. Each group significantly increased the self-perceived stress during the stress session (T1-T2), thus assuring that both groups achieved approximately the same level of self-perceived stress before the beginning of stimulation at T2. The latter also means that the MIST session attained its goal. Likewise, each group decreased their self-perceived level of stress during the relaxation session with significant differences between T2 and T3. It is a fact that reduction of the stress level happens after a certain time in a time-out room with standard white light. Fig 3 shows differences between T1, T2 and T3 not as a result of our experiment, but as a proof that the stress session (MIST) and the relaxation session (the time-out room) played their roles correctly. In summary, the significant differences of self-perceived stress T1-T2 and T2-T3 corroborate that both the stress and relaxation sessions satisfactory fulfilled their respective goals. The analysis of the subjective self-perception of stress was useful to quantify the level of stress at the beginning, during and at the end of the relaxation session and validate our methodology used in the stress (MIST) and relaxation (chromoterapy room) sessions.

Finally, the fact that most of the participants (83%) reported that the blue lighting made them get significantly more relaxed that the conventional white is a clear indication of the advantage of the use of blue lighting.

### Frontal relative gamma and heart rate

Fig 4 shows the normalized mean spectral power of Gamma, 1/Theta, 1/Alpha and the RG. These spectral bands have been used in literature to assess the level of stress [27–29]. The four plots present similar curves and they all show the drastic decrement of stress level during B1. The RG (left-bottom plot of Fig 4) is a combination of the others (see section EEG signals) that has recently used in studies relatives to meditation-relaxation [30,31] and stress [32]. The four plots exhibit a high level of correlation and a similar envelope; however the RG computes a smoother version that emphasizes the differences of curves of G1 and G2 during the transition to B1. This, together with the fact that the RG generally correlates with the HR, which is a commonly accepted stress marker [33–37], supports the use of the RG to measure the level of stress.

According to results of the linearized RG (shown in Fig 5 bottom) and the zero-crossing analysis reported in Table 2, all participants were stressed by the MIST (Seg1). Then the participants of G1 (test group), who experienced the blue lighting in B1, got the minimum level of stress approximately 1.1 minutes after the beginning of the block. However, for G2 (control group), who experienced white lighting in B1, got relaxed after approximately 3.5 minutes from the beginning of B1. The levels of stress G1-G2 measured at 1 minute after the beginning of relaxation session are significantly different (see Fig 6, minute 7). Therefore, the participants who were exposed to blue light achieved their minimum level of stress in the third part of time compared with the ones who stayed with white light. Indeed, the slope of Seg2 of G1 was significantly different of that of G2. It was approximately three-fold the slope of Seg2 of G2 (see Fig 5 bottom and Table 1, third row), thus indicating a faster acceleration of the relaxation process with blue lighting. In addition, the participants exposed to blue lighting during B1 kept the minimum level of stress for much longer time (total length of Seg3 of G1) than participants exposed to conventional white (only the initial time of Seg3).

The upper plot of Fig 5 also shows a convergence of the RG curve of both groups after 3.5–5 minutes in B1. Afterwards, the values of RG of both groups increased without significant difference (Seg4 and Seg5 in Fig 5 bottom). This fact is interpreted as follows: i) after a period of time (4 minutes approximately), there is no advantage in the use of blue lighting in comparison with the conventional white. Although the discussion about the physiological mechanism that justifies this finding is out of the scope of this work, we suggest that the sensory adaptation [46] and the tedious nature of the task could increase the level of stress; ii) then, after the convergence time (3.5–5 minutes), extended exposition to either blue or white lighting causes no additional benefit.

Fig 7 shows the HR (upper plot) and, in a similar way to the analysis performed with the RG, the linearized version (bottom plot). The linearized HR suggests that the participants experienced four phases during the experiment, one per segment. The first one (Seg1) was due to the stressful effect of the MIST. The second one (Seg2) was a consequence of the beginning of the relaxation session. These two phases corresponded to the two first phases described by the RG. The third phase (Seg3) indicated a stabilization of the stress level, that is, once the minimum was achieved, it remained low for the rest of B1. The last phase (Seg4) was similar to the previous one.

In view of Fig 7 we can state that some of the light-color-related differences indicated by the RG curves during the relaxation process cannot be observed with the HR curves. Despite the HR is generally accepted as stress marker, it has some limitations in terms of temporal resolution. In order to minimize error, the HR is usually computed through long epochs (from one to several minutes) of signal in comparison with the RG (a few seconds). In fact, we used 90-second epochs with 66% overlap and 2-second epochs without overlap for the HR and the RG, respectively. This prevents the HR from providing significant short-term differences. However, in this work, differences indicated by the RG were brief and presented at the very beginning of the relaxation session. Therefore the RG provided short-term differences in stress level that the HR was not able to highlight. In addition, we suggest that not all the neuro processes cause changes in the cardiovascular physiology. Sometimes they do affect the cardiovascular system but the changes are camouflaged with other factors that cause more powerful changes. For example, when someone is running the HR is high compared with the resting HR, but nonetheless this person may be less mentally stressed than in resting state. In the context of this paper, changes in mental stress at the beginning of the relaxation session were reflected by the RG, but they could not be indicated by the HR probably due to the full relaxed position of the participants.

### Blue vs. white lighting

In this paper we have shown that blue lighting accelerates the post-stress relaxation in comparison with conventional white. We have performed objective measures with well-known standardized procedures. In the chromoterapy room, white lighting was produced as the combination of the three sets of LEDs (red, green and blue). However, the blue one was obtained as the suppression of red and green LEDs ceteris paribus. The set of blue LEDs was the only light source in common during the whole relaxation session and paradoxically, whatever differences found in the comparison blue-white cannot be due to this wavelength, but the absence of green and red. In this sense our main claim is stated in the title of this paper and our main contribution must be understood in practical terms. The research of the influence of red or green in the level or stress is out of the scope of this study. An alternative experimental design would be to present the white light with the same luminance as the blue light. This would allow testing whether the wavelength makes a difference. However, this alternative implies different intensities of blue in each condition, together with the fact of having different color components. In this case, the analysis might be more confusing.

The fact that blue lighting accelerates the post-stress relaxation seems to be heading in the opposite direction from previous works related to melatonin suppression, sleepiness reduction and alertness augmentation [4,5,13,14,47]. Nevertheless, there are several fundamental differences between these works and our study that can explain the controversy. First of all, we analyzed post-stress relaxation instead of sleep disturbances. Secondly, the stimulus used in sleep-related works is different. Finally, the exposure time is rather short in our study. Despite that, physiological and psicological mechanisms underlying the influence of color on human beings are out of the scope of this study.

### Practical implications and future works

The findings of this work could be useful in clinical and educational environments. Psychologists and other experts that use lighting in their therapies could benefit from them. For instance, the time spent in the time-out room used in schools in episodes of violence outbreaks, can be reduced drastically to just one minute and extended for three more minutes if blue lighting is used instead of the conventional white. This would report a direct benefit to the student, who could quickly reintegrate with the rest of classmates without sense of guilt or shame and minimum impact in the training.

Furthermore, whatever color is used in the time-out room, we have shown that more than circa four minutes causes no extra benefit (potentials causes have been suggested in section Frontal relative gamma and heart rate). Previous color-lighting-related studies [19,20] spent much longer sessions of 10–15 and 30 minutes respectively. We have reported a reproducible methodology that, perhaps, will optimize results of new experiments with much shorter sessions. Obviously the results obtained in this study with healthy participants cannot be directly extrapolated to patients or students with behavioral or emotional disorders, but we have provided an easy methodology that can be applied individually to each subject and context.

Only twelve volunteers participated in the study. This sample is not large enough to obtain solid scientific evidence but, as a first approach, it may establish the pillars for future studies. The paper reports the methodology and results of a preliminary study that can motivate further research in the field. Our results must be extended to draw reliable conclusions. Despite that, the statistics revealed promising results that are relevant for the scientific community.

Apart from that, the information reported here could influence in emerging technologies such as neuromarketing (e.g., the use of a blue lighting for a short while just before starting a negotiation) and in daily-life context (e.g., during stressful periods of work or at home). Stress has an important role in people life and this preliminary work might be used as a source to investigate stress-color relationship through an accurate methodology based on bio-signals.

## Supporting information

S1 File Study protocol and bioethics committee endorsement. (ZIP)

S2 File Datasets of subject 1. (ZIP)

S3 File Datasets of subject 2. (ZIP)

S4 File Datasets of subject 3. (ZIP)

S5 File Datasets of subject 4. (ZIP)

S6 File Datasets of subject 5. (ZIP)

S7 File Datasets of subject 6. (ZIP)

S8 File Datasets of subject 7. (ZIP)

S9 File Datasets of subject 8. (ZIP)

S10 File Datasets of subject 9. (ZIP)

S11 File Datasets of subject 10. (ZIP)

S12 File Datasets of subject 11. (ZIP)

S13 File Datasets of subject 12. (ZIP)
