# Supplementary material for: Blue lighting accelerates post-stress relaxation: Results of a preliminary study
Source: PLoS One. 2017 Oct 19;12(10):e0186399. doi: 10.1371/journal.pone.0186399 (PMC5648169; doi:10.1371/journal.pone.0186399)
Supplement: S1 File — (ZIP) [file pone.0186399.s001.zip › bioethics_committee.pdf]

COMITE DE ETICA EN INVESTIGACION DE LA UNIVERSIDAD DE GRANADA

La Comisión de Ética en Investigación de la Universidad de Granada, analizado el informe preliminar del Presidente del Comité en Investigación Humana, emite informe favorable a la metodología en la investigación titulada 'ESTUDIO DE LOS EFECTOS DE LA CROMOTERAPIA MEDIANTE ANáLISIS DE BIO-SEñALES (PARA EL PROYECTO DE EXCELENCIA DE LA JA: PLAT-EEG, REF. P11-TIC-7983)' que dirige D./Dña. FRANCISCO JOSÉ PELAYO VALLE, con NIF 74.617.146-V, quedando registrada con el nº: 183/CEIH/2016.

Granada, a 05 de Mayo de 2016.

UNIVERSIDAD DE GRANADA
VICERRECTORADO DE INVESTIGACIÓN Y TRANSFERENCIA

F. Cornet

EL PRESIDENTE
Fdo: Enrique Herrera Viedma

EL SECRETARIO
Fdo: Fernando Cornet Sánchez del Águila
