# Supplementary material for: Blue lighting accelerates post-stress relaxation: Results of a preliminary study
Source: PLoS One. 2017 Oct 19;12(10):e0186399. doi: 10.1371/journal.pone.0186399 (PMC5648169; doi:10.1371/journal.pone.0186399)
Supplement: S1 File — (ZIP) [file pone.0186399.s001.zip › informed_consent.pdf]

CONSENTIMIENTO INFORMADO

Estudio de los efectos de la cromoterapia mediante análisis de bio-señales

Responsable del estudio: Prof. Dr. Francisco José Pelayo Valle

- Tratamos de hacer que este formulario sea fácil de entender pero puede que todavía contenga ideas o palabras que no le parezcan claras. Por favor, siéntase con confianza de pedirle al investigador o al personal técnico relacionado con el estudio que le explique cualquier cosa que usted no entienda.
- Si está interesado en participar como voluntario en este estudio por favor lea el documento de "Información del procedimiento" y complete el siguiente cuestionario.

A rellenar por el investigador:

Fecha de realización del estudio:

Lugar de realización del estudio y máquina de registro de bio-señales: Sala de cromoterapia del Colegio de Educación Especial "Clínica San Rafael", en Granada. Miniature Data Acquisition System de Cognionics, Inc. (San Diego, USA).

Otras observaciones (ANOTAR AQUÍ NÚMERO DE PARTICIPANTE):

Datos personales del participante:

Nombre y apellidos:	DNI:
Fecha de nacimiento:	Edad:
Domicilio:	Código Postal:
Localidad:	Provincia:
Teléfono de contacto:	E-mail:

Información para la realización de la tarea:

Patologías visuales:

¿Ha participado alguna vez en un experimento de EEG? […]   ¿Y de cromoterapia? [...]

¿Padece algún tipo de fobia a espacios cerrados y/o pequeños?

¿Padece algún tipo de enfermedad/patología/alergia relacionada con la luz?

Otras observaciones que puedan afectar al desenvolvimiento del estudio:

Dentro del marco este experimento y bajo las condiciones del consentimiento informado que firmo, AUTORIZO a que se registren fotos y vídeos míos durante el desarrollo del mismo.

Certifico que este formulario me ha sido explicado en su totalidad y que lo entiendo, o me fue leído a mí y lo he entendido. También entiendo los riesgos y peligros que se presentan y estoy lo suficientemente informado para dar mi consentimiento. Entiendo el documento de información del procedimiento que contiene una descripción completa de los usos y divulgación de mi información personal y de salud y de mis registros EEG y ECG.

Nombre del participante:

Lugar, fecha:

Firma:

Firma del investigador responsable:

Fdo. Francisco José Pelayo Valle

……………………………………………………………………………………………...

Certificado de la persona que obtiene el consentimiento. Se ha informado al voluntario de:

i) El procedimiento, propósito y riesgos del estudio como se describió con anterioridad.
ii) Como podrá usarse, compartirse y reportarse su información de salud.
iii) Sus derechos de privacidad y de renuncia al proyecto.

Y para que así conste firma el presente consentimiento.

Nombre:

Lugar, fecha:

Firma:
