# Supplementary material for: Blue lighting accelerates post-stress relaxation: Results of a preliminary study
Source: PLoS One. 2017 Oct 19;12(10):e0186399. doi: 10.1371/journal.pone.0186399 (PMC5648169; doi:10.1371/journal.pone.0186399)
Supplement: S1 File — (ZIP) [file pone.0186399.s001.zip › procedure_information.pdf]

INFORMACIÓN DEL PROCEDIMIENTO

Estudio de los efectos de la cromoterapia mediante análisis de bio-señales.

Responsable del estudio: Prof. Dr. Francisco José Pelayo Valle

- De acuerdo con el artículo 15 de la Ley 14/2007, de 3 de Julio, de Investigación biomédica a continuación le presentamos toda la información relativa al procedimiento y características de este estudio de investigación.
- Le ofrecemos la oportunidad de participar en este estudio. Estamos estudiando los efectos de la cromoterapia sobre el nivel de relajación en sujetos sanos. Tras obtener testimonios del efecto relajante del uso de luz con ciertos colores en niños con discapacidad intelectual y trastornos de conducta y en niños con discapacidad motora, nos disponemos a registrar y analizar diferentes bio-señales para respaldar, primero en sujetos sanos y mediante medidas objetivas, las evidencias encontradas a través de valoraciones subjetivas. La investigación incluye realización de registros de electroencefalograma (EEG) y electrocardiograma (ECG). Previo al trabajo con la cromoterapia, se realizarán una serie de actividades cognitivas para asegurar el funcionamiento del experimento.
- La técnica del EEG se desarrolló a principios del siglo XX y desde entonces se utiliza en todo el mundo, llegando a ser una herramienta muy útil tanto en la clínica como en la investigación. Se trata de una técnica que no es ni invasiva ni dolorosa. Unos electrodos serán convenientemente fijados en distintas posiciones de su cabeza para el registro del EEG, con el concurso de un gel conductor y algún sistema de fijación (ventosa, velcro, gorro, etc.). Previamente la zona será limpiada con un gel especial para eliminar las células muertas más externas de la piel, mejorando así la calidad de la señal EEG a registrar. El proceso descrito es simple, rápido e indoloro, no causa molestia alguna y es completamente seguro. Se han realizado numerosos estudios con esta técnica y se ha demostrado la carencia de efectos secundarios o consecuencias indeseables en humanos si se siguen las pautas de actuación adecuadas.
- La técnica del ECG tiene su origen a finales del siglo XIX y desde entonces se utiliza en todo el mundo. Como el EEG, el ECG se convirtió en una herramienta muy útil tanto en el ámbito clínico como en investigación. Se trata de una técnica no invasiva y no dolorosa. Uno o varios electrodos serán convenientemente fijados a la piel en una o varias posiciones de su cuerpo para el registro del ECG mediante el uso de parches adhesivos. El proceso descrito es simple, rápido e indoloro, no causa molestia alguna y es completamente seguro. Se han realizado numerosos estudios con esta técnica y se ha demostrado la carencia de efectos secundarios o consecuencias indeseables en humanos si se siguen las pautas de actuación adecuadas.
- La cromoterapia o terapia de los colores es una técnica de medicina alternativa basada en la estimulación con fuentes de luz de distintas longitudes de onda, que percibimos como colores diferentes. Mediante iluminación puntual o ambiental de un color, se persigue influenciar el estado emocional del participante.
- Todas las técnicas mencionadas no tienen contraindicaciones ni efectos negativos. No se aplica ningún tipo de estimulación susceptible de causar daños. Además, la instrumentación para la toma de registros carece de conexión a la red eléctrica. La única molestia que se podría ocasionar es la necesidad de limpiar con agua y jabón los restos de gel del pelo. Hay que seguir algunas recomendaciones que le indicará el técnico responsable. Usted en todo momento de la prueba podrá comunicarse con los técnicos, pudiendo parar el procedimiento en cualquier instante. Con mucho gusto hablaremos con usted legal acerca de cualquier duda que tenga.

- El estudio se realizará en la sala de cromoterapia del Colegio de Educación Especial "Clínica San Rafael", en Granada.
- Este estudio busca evidencias acerca del efecto tranquilizante de la cromoterapia.
- El experimento constará de una sesión por participante, de una duración aproximada de 45 minutos. Este tiempo estará dividido en dos partes, después de la preparación. Una primera para realizar una serie de tareas cognitivas y la segunda, dividida a su vez en dos bloques de 10 minutos cada uno, en donde el participante se enfrentará a sesiones de luz de un color e intensidad determinados. Durante este tiempo, el participante deberá estar en la sala, sin mirar a ningún punto en concreto, sentado cómodamente sobre un puf blanco.
- Deberá dejar fuera de la sala todo material excepto aquél necesario para el registro de bio-señales y la indumentaria que le sea suministrada por el personal técnico, la cual deberá vestir durante la realización del experimento. Ésta consistirá en un uniforme blanco tipo hospital.
- El estudio se realizará en una sola sesión. En algunos participantes se valorará la posibilidad de realizar un segundo estudio si es pertinente y éste accede.
- Durante el estudio se le monitorizará mediante una cámara de vídeo. Esto se hará para asegurar que el participante realiza el experimento de manera correcta, además de para asegurar el bienestar de la persona. Además de grabar, se podrán tomar fotos, previo aviso y consentimiento del participante. Todos estos datos se tomarán para su posterior uso en investigación y eventual publicación de los resultados. Todo se hará cumpliendo la normativa existente, la Ley Orgánica 15/1999, de 13 de diciembre, de Protección de Datos de Carácter Personal.
- Al principio y al final del experimento Ud. deberá rellenar un cuestionario.
- El estudio se hace únicamente por motivo de I+D+i.
- El equipo que realiza el estudio recogerá toda la información en un archivo en el que usted no podrá ser identificado. Los datos así guardados serán estrictamente confidenciales y se seguirán las normas adecuadas según lo recogido por la legislación vigente acerca de la autonomía del paciente y de los derechos y obligaciones en materia de información y documentación clínica así como la protección de datos de carácter personal (Ley 14/2007, de 3 de julio, de Investigación biomédica, Ley 41/2002, de 14 noviembre, y la Ley Orgánica 15/1999, de 13 de diciembre).
- El estudio está financiado por el proyecto de investigación P11-TIC-7983 de la Junta de Andalucía y el proyecto de investigación TIN2012-32039 del Ministerio de Economía y Competitividad, cofinanciado con fondos FEDER, y la Asociación Asociación NICOLO para la promoción del I+D+i de Neurotecnologías y servicios en el ámbito de la discapacidad.
- Los resultados del estudio tendrán como único fin el investigador y desarrollo de herramientas y tecnologías derivadas del conocimiento generado y podrán publicarse en revistas científicas especializadas, exponerse en congresos especializados, presentarse a autoridades sanitarias, científicas o académicas, o explotarse a través de patentes, licencias de explotación o comercialización directa. En ningún caso figurará su nombre o cualquier otro dato que pueda identificarle.
- No dude en plantear cualquier pregunta que pueda surgirle en relación con el estudio o con esta información. No se sienta obligado a participar si no lo desea. Si no está de acuerdo con cualquier parte de la información que aquí le hemos presentado no participe en el estudio. En caso de que decida participar puede interrumpir su colaboración en el momento

que lo desee. En ningún modo esta decisión repercutirá en el trato que reciba. Además, su participación o la negativa a participar en él no tendrán repercusión alguna.

- Usted podrá solicitar en cualquier momento que la información recogida durante el experimento no sea considerada. En ese caso borraremos todos los registros e información derivada de su participación, siendo estos excluidos del estudio.
- Si después de intervenir en el estudio le surge cualquier duda o pregunta, estaremos a su completa disposición. Puede contactar con:

Prof. Dr. Francisco José Pelayo Valle
Departamento de Arquitectura y Tecnología de Computadores
Despacho 35, 2ª planta, E.T.S. Ingenierías Informática y de Telecomunicación
C/ Periodista Daniel Saucedo Aranda, S/N, 18071 Granada
Email: fpelayo@ugr.es
Teléfono: 958240581

- Usted, como participante, manifiesta expresamente que la respuesta a todas las preguntan que se le formularán serán reales.
- Por su participación en el experimento, no recibirá ningún tipo de compensación económica. Además, se le informa que el registro de las muestras se realiza de manera gratuita, donando usted las mismas.
- Si entiende toda la información que contiene este escrito y está dispuesto a participar en el estudio, por favor pase a rellenar el documento “Consentimiento informado”.
