# Supplementary material for: Blue lighting accelerates post-stress relaxation: Results of a preliminary study
Source: PLoS One. 2017 Oct 19;12(10):e0186399. doi: 10.1371/journal.pone.0186399 (PMC5648169; doi:10.1371/journal.pone.0186399)
Supplement: S1 File — (ZIP) [file pone.0186399.s001.zip › study_protocol_english.pdf]

Study protocol (english version)

1. Materials

The following materials must be checked before starting the experiment:

- 10-20 International System EEG cap (electrodes attached to cap).
- Measuring tape.
- Harness to fix EEG cap.
- Ear clip EEG electrode for reference and ground.
- ECG electrode.
- 16-channel data acquisition system (Miniature Data Acquisition System of Cognionics, Inc.).
- Conductive gel for EEG electrodes.
- Syringe and needle.
- Impedance measurement equipment (EIM 105-30HZ of General Devices, Inc.).
- Laptop 1 for biosignals recording.
- Laptop 2 for Montreal Imaging Stress Task (MIST).
- White hospital uniform.
- Wavelength and luminance measurement equipment (i1 Display Pro of X-Rite, Inc.).
- Preparation room with a comfortable chair.
- Chromotherapy room (6 m^2) with a comfortable puff-shaped seat, a video camera and a lighting system consisting of three sets of light-emitting diodes (LEDs): red (616 nm wavelength and 2.19 cd/m^2 luminance), green (550 nm wavelength and 4.02 cd/m^2 luminance) and blue (471 nm wavelength and 1.37 cd/m^2 luminance) LEDs. Check these parameters by using the wavelength and luminance measurement equipment.

2. Methodology

Methods described in this section must be carefully followed by the researcher in order to ensure the integrity and reproducibility of the study.

2.1 Initial procedure

First of all, the researcher must inform the participant of the experimental procedure and give him/her the opportunity to drop out of the study at any moment. The researcher must provide a document containing information about the experiment. This document together with the informed consent must be read by the participant. Once the informed consent is understood, accepted and signed by the participant, the researcher must record the number of participant and the corresponding group: G1 (blue-white) or G2 (white-blue).

2.2 Participant preparation

The researcher must prepare the participant for the experiment (within the preparation room) by following the next instructions:

- Clothe the participant with the white hospital uniform, the EEG cap, the harness, the ear clip EEG electrode and the ECG electrode (non-dominant wrist of the participant).
- Ensure that Cz electrode of the EEG cap is placed in the middle of the line from the inion to the nasion. Use the measuring tape for that.
- Attach the EEG cap and the data acquisition system to the harness.
- Use the syringe and the needle to put conductive gel at the following electrode positions: Fp1, Fp2, Fz, F3, F4, F7, F8.
- Ensure that the impedance of every single electrode is below 30 KΩ. Use the impedance measure equipment for that.

- Connect the cited electrodes to the data acquisition system.
- Use laptop 1 for real-time visualization of signals. Check the morphology, amplitude and timing of the signals by visual inspection.
- If morphology, amplitude and timing of the signals are within normal ranges, start signal recording by using laptop 1.

2.3 Experimental procedure

Every single event of the experiment (e.g., beginning and end of a phase) must be recorded by using a trigger marker in recorded raw data.

Before the stress session, the researcher must record the answers to the questions of the test based on the Perceived Stress Scale (PSS) (1).

Afterwards, the stress session starts. The researcher must explain the MIST to the participant. The MIST must be conducted within the preparation room. The participant must use the graphical user interface (GUI) of the MIST implemented in Matlab R2014a. During the task, the participant must sit on a chair and try to exclusively move his/her dominant hand using the touchpad (in order to avoid severe artifacts in EEG and ECG signals). The training period must last 3 minutes and the MIST must last 6 minutes. Guidelines described in (2) must be rigorously followed to ensure success in the stress session.

After the stress session and before the relaxation session, the researcher must record the answers to the questions of the test based on the Perceived Stress Scale (PSS) (1) for the second time.

Afterwards, the relaxation session starts. The researcher must explain the relaxation session to the participant. The participant must stay laid on the comfortable puff-shaped seat placed inside the chromotherapy room. The participant must be instructed not to close their eyes (except for blinking) and to avoid moving or gazing any part of the room (i.e., the thousand-yard stare) during the relaxation session. During the stay, the participant must be monitored by the video camera for safety and artifacts removal purposes. The researcher (located in the preparation room) must monitor the participant and record every relevant event. The relaxation session must last 20 minutes. The first 10 minutes correspond to the first block (B1). In this block, the chromotherapy room is lighted with blue light (by powering up the blue LEDs with red and green LEDs powered down) for participants of G1. The second 10 minutes correspond to the second block (B2). In this block, the chromotherapy room is lighted with white light (by powering up all the LEDs) for participants of G1. For participants of G2, the light sequence is the opposite (i.e., white-blue).

After the relaxation session, the researcher must record the answers to the questions of the test based on the Perceived Stress Scale (PSS) (1) for the third time. This time, the test includes the following extra question: *Which color, blue or white, have you felt more relaxed with?*

Afterwards, the researcher must help the participant to remove the EEG cap, ECG electrode, the ear clip EEG electrode, the harness and the hospital uniform. The researcher must provide the participant with the necessary products to clean his/her hair.

Finally, the researcher must thank the participant for his/her participation in the study and record some feedback.

References

1. Remor E. Psychometric Properties of a European Spanish Version of the Perceived Stress Scale (PSS). Span J Psychol [Internet]. Cambridge University Press; 2014 Apr 10 [cited 2016 Feb 19];9(01):86–93. Available from: http://journals.cambridge.org/abstract_S1138741600006004

2. Dedovic K, Renwick R, Mahani NK, Engert V, Lupien SJ, Pruessner JC. The Montreal Imaging Stress Task: using functional imaging to investigate the effects of perceiving and processing psychosocial stress in the human brain. J Psychiatry Neurosci [Internet]. 2005;30(5):319–25. Available from: http://www.pubmedcentral.nih.gov/articlerender.fcgi?artid=1197276&tool=pmcentrez&rendertype=abstract
