# Supplementary material for: Blue lighting accelerates post-stress relaxation: Results of a preliminary study
Source: PLoS One. 2017 Oct 19;12(10):e0186399. doi: 10.1371/journal.pone.0186399 (PMC5648169; doi:10.1371/journal.pone.0186399)
Supplement: S1 File — (ZIP) [file pone.0186399.s001.zip › study_protocol_original.pdf]

Protocolo del estudio (original version)

1. Materiales

Los materiales de la siguiente lista deben ser comprobados por el investigador antes del inicio del experimento:

- Gorro EEG del sistema internacional 10-20 (electrodos incluidos en el gorro).
- Cinta métrica.
- Arnés para fijación del gorro EEG.
- Electrodo EEG de oreja tipo pinza para tierra y referencia.
- Electrodo ECG.
- Sistema de adquisición de datos de 16 canales (Miniature Data Acquisition System de Cognionics, Inc.).
- Gel conductivo para electrodos EEG.
- Jeringa y aguja.
- Equipo de medida de impedancias (EIM 105-30HZ of General Devices, Inc.).
- Ordenador portátil 1 para grabación de bioseñales.
- Ordenador portátil 2 para el Montreal Imaging Stress Task (MIST).
- Uniforme de hospital blanco.
- Equipo de medida de longitud de onda y luminancia (i1 Display Pro of X-Rite, Inc.).
- Habitación de preparación con una silla cómoda dentro.
- Habitación de cromoterapia (6 m^2) con un asiento cómodo tipo puff, una cámara de vídeo y un sistema de iluminación compuesto por tres conjuntos de electrodos emisores de luz (LEDs): rojos (longitud de onda de 616 nm y luminancia de 2.19 cd/m^2), verdes (longitud de onda de 550 nm y luminancia de 4.02 cd/m^2) y azules (longitud de onda de 471 nm y luminancia de 1.37 cd/m^2). Estos parámetros deben ser comprobados con el equipo de medida de longitud de onda y luminancia.

2. Metodología

La metodología descrita en esta sección debe ser meticulosamente seguida por el investigador para asegurar la integridad y reproducibilidad del estudio.

2.1 Procedimiento inicial

Antes de todo, el investigador debe informar al participante sobre el procedimiento experimental y darle la oportunidad de abandonar en cualquier momento. El investigador debe dar al participante el documento con la información del procedimiento. Junto con este documento, el consentimiento informado debe ser leído, comprendido, aceptado y firmado por el participante. Una vez finalizado este proceso, el investigador debe recoger el número de participante y el grupo correspondiente al que pertenece: G1 (azul-blanco) o G2 (blanco-azul).

2.2 Preparación del participante

El investigador debe preparar al participante (dentro de la habitación de preparación) siguiendo las siguientes instrucciones:

- Vista al participante con el uniforme de hospital blanco (encima de su ropa), el gorro EEG, el arnés de fijación, el electrodo EEG tipo pinza para la oreja y el electrodo ECG (colocado en la muñeca de la mano no dominante del participante).
- Asegúrese que el electrodo correspondiente a la posición Cz del gorro queda dispuesto en la mitad de la línea que une los huesos inion y nasion. Use la cinta métrica para ello.
- Enganche el gorro EEG y el sistema de adquisición de datos al arnés de fijación.

- Use la jeringa y la aguja para introducir gel conductivo en las siguientes posiciones de electrodo: Fp1, Fp2, Fz, F3, F4, F7, F8.
- Asegúrese que la impedancia de cada electrodo es menor que 30 KΩ. Use el equipo de medida de impedancias para ello.
- Conecte los electrodos citados al sistema de adquisición de datos.
- Use el ordenador portátil 1 para la visualización en tiempo real de señales. Compruebe la morfología, amplitud y evolución temporal de estas señales mediante inspección visual.
- Si la morfología, amplitud y evolución temporal de las señales están dentro de los rangos normales, inicie la grabación de señales usando el ordenador portátil 1.

2.3 Procedimiento experimental

Cada evento del experimento (e.g., inicio y final de cada fase) debe ser recogido y marcado mediante un marcador de disparo en los datos raw.

Antes de la sesión de estrés, el investigador debe recoger las respuestas al cuestionario basado en el Perceived Stress Scale (PSS) (1).

A continuación comienza la sesión de estrés. El investigador debe explicar el MIST al participante. El MIST debe ser llevado a cabo dentro de la habitación de preparación. El participante debe usar la interfaz gráfica de usuario (GUI) del MIST implementada en Matlab R2014a. Durante la tarea, el participante debe permanecer sentado en una silla e intentar mover únicamente su mano dominante para el uso del touchpad, con objeto de evitar artefactos bruscos en las señales de EEG y ECG. El período de entrenamiento del MIST debe durar 3 minutos, mientras que el MIST debe durar 6 minutos. Las pautas descritas en (2) deben ser estrictamente seguidas para asegurar el objetivo de la sesión de estrés.

Después de la sesión de estrés y antes de comenzar la sesión de relajación, el investigador debe, por segunda vez, recoger las respuestas al cuestionario basado en el Perceived Stress Scale (PSS) (1).

A continuación comienza la sesión de relajación. El investigador debe explicar la sesión de relajación al participante. El participante debe permanecer tumbado en el asiento tipo puff situado dentro de la habitación de cromoterapia. El participante debe ser instruido para no cerrar los ojos (excepto para parpadear), evitar moverse y evitar mirar a ningún punto concreto de la habitación durante la sesión. El participante debe ser monitorizado mediante la cámara de vídeo con objeto de garantizar su seguridad y para identificación de posibles artefactos. El investigador, situado en la habitación de preparación debe observar al participante y recoger cualquier evento relevante. La sesión de relajación debe durar 20 minutos. Los primeros 10 minutos corresponden al primer bloque (B1). En este bloque, la habitación de cromoterapia es iluminada con luz azul (encendiendo todos los LEDs azules con el resto apagados) para los participantes del G1. Los segundos 10 minutos corresponden al segundo bloque (B2). En este bloque, la habitación de cromoterapia es iluminada con luz blanca (encendiendo todos los LEDs azules, rojos y verdes) para los participantes del G1. Para los participantes del G2, la secuencia de luces es la opuesta (i.e., blanco-azul).

Después de la sesión de relajación, el investigador debe, por tercera vez, recoger las respuestas al cuestionario basado en el Perceived Stress Scale (PSS) (1). Esta vez, el test incluye una pregunta extra: *¿Con qué color te has sentido más relajado?*

A continuación, el investigador debe ayudar al participante a quitarse el gorro EEG, el electrodo ECG, el electrodo EEG tipo pinza, el arnés de fijación y el uniforme de hospital. El investigador debe ofrecer al participante los productos necesarios para que limpie su cabello.

Finalmente, el investigador debe agradecer al participante por su participación y recoger

algún feedback.

Referencias

1. Remor E. Psychometric Properties of a European Spanish Version of the Perceived Stress Scale (PSS). Span J Psychol [Internet]. Cambridge University Press; 2014 Apr 10 [cited 2016 Feb 19];9(01):86–93. Available from: http://journals.cambridge.org/abstract_S1138741600006004
2. Dedovic K, Renwick R, Mahani NK, Engert V, Lupien SJ, Pruessner JC. The Montreal Imaging Stress Task: using functional imaging to investigate the effects of perceiving and processing psychosocial stress in the human brain. J Psychiatry Neurosci [Internet]. 2005;30(5):319–25. Available from: http://www.pubmedcentral.nih.gov/articlerender.fcgi?artid=1197276&tool=pmcentrez&rendertype=abstract
